# Circulating microRNAs miR-331 and miR-195 differentiate local luminal a from metastatic breast cancer

**DOI:** 10.1186/s12885-019-5636-y

**Published:** 2019-05-10

**Authors:** Peter McAnena, Kahraman Tanriverdi, Catherine Curran, K. Gilligan, Jane E. Freedman, James A. L. Brown, Michael J. Kerin

**Affiliations:** 1 Discipline of Surgery, Lambe Institute for Translational Research, School of Medicine, National University of Ireland Galway, Galway, Ireland; 2 UMass Memorial Heart & Vascular Center, University of Massachusetts Medical School, The Albert Sherman Center, 7th Floor West, AS7-1051, 368 Plantation St, Worcester, MA 01605-4319 USA

**Keywords:** miRNA, Breast, Cancer, Biomarker, Metastasis, miR-331, miR-195

## Abstract

**Background:**

Breast cancer is the leading cause of cancer related death in women, with metastasis the principle cause of mortality. New non-invasive prognostic markers are needed for the early detection of metastasis, facilitating treatment decision optimisation. MicroRNA (miRNA) are small, non-coding RNAs regulating gene expression and involved in many cellular processes, including metastasis. As biomarkers, circulating miRNAs (in blood) hold great promise for informing diagnosis or monitoring treatment responses.

**Methods:**

Plasma extracted RNA from age matched local Luminal A (*n* = 4) or metastatic disease (n = 4) were profiled using Next Generation Sequencing. Selected differentially expressed miRNA were validated on a whole blood extracted miRNA cohort [distant metastatic disease (*n* = 22), local disease (*n* = 31), healthy controls (*n* = 21)]. Area Under the Curve (AUC) in Receiver Operating Characteristic (ROC) analyses was performed.

**Results:**

Of 4 miRNA targets tested (miR-181a, miR-329, miR-331, miR-195), mir-331 was significantly over-expressed in patients with metastatic disease, compared to patients with local disease (*p* < 0.001) or healthy controls (p < 0.001). miR-195 was significantly under-expressed in patients with metastatic disease, compared to patients with local disease (p < 0.001) or healthy controls (*p* = 0.043). In combination, miR-331 and miR-195 produced an AUC of 0.902, distinguishing metastatic from local breast cancer.

**Conclusions:**

We identified and validated two circulating miRNAs differentiating local Luminal A breast cancers from metastatic breast cancers. Further investigation will reveal the molecular role of these miRNAs in metastasis, and determine if they are subtype specific. This work demonstrates the ability of circulating miRNA to identify metastatic disease, and potentially inform diagnosis or treatment effectiveness.

**Electronic supplementary material:**

The online version of this article (10.1186/s12885-019-5636-y) contains supplementary material, which is available to authorized users.

## Background

Breast cancer is the most common cancer among women and the fifth leading cause of cancer death, with metastasis the principle cause of mortality [1]. Despite considerable recent advances in both diagnosis and treatment 20–30% of breast cancer patients will present with, or develop, distant metastatic disease [2]. The risk of developing metastatic disease is determined by the initial stage at detection, as well as tumour subtype and access to appropriate therapy. Breast cancer consists of at least four clinically relevant molecular subtypes: Luminal A, Luminal B, HER2-enriched and triple-negative [3]. Luminal A is the most common subtype, comprising up to 60% of all breast cancers [4, 5]. Bone is the most frequent site of metastasis among all subtypes, with a recent study (in > 240,000 cases) showing that 58.52% of metastasis in Luminal A was to the bone, with an incidence of 3.1% [6]. Luminal A patients remain at considerable risk of metastasis after 5 years in contrast to triple-negative patients, who tend to develop metastases in the first 3 years following diagnosis [7]. New, non-invasive, biomarkers capable of augmenting conventional diagnostic and prognostic modalities in metastatic breast cancer are the key to facilitating truly individualised treatment (and disease monitoring) regimens.

MicroRNAs (miRNA) are small, non-coding RNAs that regulate gene expression by targeting messenger RNA, resulting in either translational repression or RNA degradation [8]. Over 4000 miRNAs have been described, and it is estimated that they regulate up to 30% of all human genes [9]. MiRNAs can operate as tumour-suppressors or tumour-promoters and their dysregulation is intricately linked to cellular processes involved in the metastatic cascade, such as sustained proliferation, angiogenesis and epithelial-mesenchymal transition (EMT) [10, 11]. Circulating miRNAs show great promise in contributing to the diagnosis, prognosis, evaluation of response to therapy and treatment of breast cancer [12–15]. MiRNAs are stable in circulation and can be quantified relatively simply and inexpensively (using real-time quantitative reverse transcriptase PCR; RT-qPCR) [16–18].

The aim of our study was to identify and validate circulating miRNAs capable of distinguishing metastatic breast cancer from locally confined breast cancer in Luminal A patients. We utilised Next Generation Sequencing (NGS) to profile circulating miRNAs, with selected differentially expressed miRNAs validated in an independent cohort (including healthy controls). Building on our previous work which identified mir-195 as a circulating biomarker in breast cancer [16], we further investigated if miR-195 was a biomarker for Luminal A or metastasis. Additionally, the selected target miRNA were combined and tested as part of a miRNA signature, for an improved ability to identify metastatic disease.

## Methods

### Patient selection

Samples were selected from a prospectively maintained Biobank (2008–2015) collected from breast cancer patients (primarily from the west coast of Ireland) treated at a tertiary referral unit (Galway University Hospital, Galway Ireland). The discovery cohort was composed of plasma samples from patients with Luminal A disease metastasized to the bone, and patients with locally confined Luminal A breast cancer (*n* = 4) (Table 1). The validation cohort (*n* = 74) was comprised of whole blood samples from 22 patients with distant metastasized Luminal A disease (17 to bone), patients with locally confined Luminal A breast cancer (*n* = 31) and healthy age matched controls (*n* = 21) (Table 2). All breast cancer patients in the study had histologically confirmed Luminal A breast cancer; hormone receptor positive and HER2/neu negative. Receptor status was taken from routine clinical evaluation records [ER/PR status determined using immunohistochemistry as per ASCO guidelines, ALLRED score ≥ 3 (1–10% weakly positive cells). HER2 receptor status was identified by Herceptest, with a score of 3+ considered positive. Any + 2 inconclusive results were confirmed using FISH testing as per ASCO guidelines, with a HER2/CEP17 ratio greater than two considered amplified]. Metastatic patients had confirmed distant metastatic disease by biopsy/imaging or both, at the time samples were collected. Blood was obtained from the locally confined breast cancer patients pre-operatively and these patients had no evidence of subsequent recurrence or metastasis (mean follow up of 7.2 years). No patients received neo-adjuvant chemotherapy. Age-matched healthy control blood samples were collected from women residing in the same catchment area as the cancer cases. Women providing control samples were interviewed by a clinician in advance of sample collection, confirming there was no personal history of malignancy, or current inflammatory or infectious conditions.

**Table 1:** Discovery cohort clinicopathological details

Clinicopathological details	Metastatic (n = 4)	Local (n = 4)
Age - mean years	61	65
Histological Subtype	Ductal (n = 4)	Ductal (n = 4)
Molecular Subtype	Luminal A (n = 4)	Luminal A (n = 4)
Stage	IV (n = 4)	II (n = 2) III (n = 2)
Metastasis location	Bone (n = 4)	–
Time Sample Taken	M1 at presentation (n = 1) Metastatic at follow up (n = 3)	Pre-operatively (n = 4)

**Table 2:** Validation cohort clinicopathological details

Clinicopathological details	Metastatic (n = 22)	Local (n = 31)	Healthy Controls (n = 21)
Age - mean years (SD)	60 (±15 years)	54 (±12 years)	52 (±12 years)
Histological Subtype	Ductal (n = 17) Lobular (n = 5)	Ductal (n = 31)	–
Molecular Subtype	Luminal A (n = 22)	Luminal A (n = 31)	–
Stage	IV (n = 22)	I (n = 10) II (n = 17) III (n = 4)	–
Tumour Grade		1 (*n* = 6) 2 (n = 17) 3 (*n* = 8)	–
Nodal Status		N positive (n = 11) N negative (n = 20)	–
Metastasis location	Bone (n = 17) Lung (n = 3) Liver (n = 2)	–	–
Time Sample Taken	M1 at presentation (*n* = 14) Metastatic at follow up (n = 8)	Pre-operatively (n = 31)	–

### RNA extraction

RNA was isolated from plasma samples (500 μl) using a miRNeasy Serum/Plasma Kit (Qiagen) and a semi-automated QIAcube platform (Qiagen), both as per the manufactures instructions. Whole blood RNA was extracted from venous non-fasting whole blood samples collected in BD vacutainers® containing 18 mg dipotassium EDTA anticoagulant (BD-Plymouth, PL6 7BP, UK). Total RNA was extracted from whole blood (1 ml) using TRI Reagent BD (Molecular Research Centre, Inc) as previously described [19]. RNA concentration was determined by NanoDrop spectrophotometry (NanoDrop ND-1000 Technologies Inc., DE, USA).

### Library preparation for RNA sequencing

Small-RNA libraries were constructed using NEXTflex Small RNA Sequencing Kit (Ion PGM & Ion Proton Compatible, Bioscientific) as per the manufactures instructions.

### Next generation sequencing

Next Generation Sequencing (maximum 200 nucleotide read) performed using the Ion PI Sequencing 200 Kit v2 (Life Technologies, USA) and Ion PI Chip Kit v2 BC as per the manufactures instructions, on the Ion Proton System (Life Technologies, USA).

### Next generation sequencing data analysis

Analysis was performed as previously described using the exceRpt pipeline [15]. Briefly, the ExceRpt pipeline [15] utilizes assesses microRNAs (miRNAs) using a series of read-alignment processes calculated to eliminate potential contaminants. Key steps include: .3′ adapter clipping (adapter removal accomplished using the fastx v.0.0.13 clipper tool; http://hannonlab.cshl.edu/fastx_toolkit/); Quality control and [removal of probable contaminant sequences using Bowtie2 and UniVec (NCBI common contaminant sequences library) and human ribosomal RNA (rRNA) precursor sequences (45S, 5S and mitochondrial rRNA sequences)]; sRNAbench software tool analysis [15] [for each sample, identical clipped-read sequences are counted and collapsed to a single entry, reads containing N’s are removed, and using Bowtie1 mapped (to the human genome and pre-miRNA sequences from miRBase v21, allowing a single mismatched base in each alignment). The alignment outcomes (pre-miRNA and mature-miRNA IDs) are parsed and used in conjunction with Vienna software]; Reads not previously aligned are mapped against small-RNA libraries (including tRNAs from gtRNAd, piRNAs from RNAdb, snoRNAs from snoRNA-LBME-db snRNAs, other RNA sequences from RFam); Finally, any outstanding unmapped reads are aligned (using sRNAbench), to the annotated plant and virus pre-miRNA sequences in miRBase (complete set).

### Validation by RT-qPCR

RT-qPCR of target miRNA was performed using TaqMan miRNA assays (Applied Biosystems). Following RNA isolation, 100 ng of total RNA was reverse transcribed using stem-loop primers and MultiScribe reverse transcriptase. PCR reactions were performed in triplicate with inter assay control (IAC) used. RQ-PCR performed using standard thermal-cycling conditions (7900HT Fast Real-Time PCR System, Applied Biosystems). Raw fluorescence (cycle threshold, C_T_) data were subsequently calculated. High C_q_ values indicated low miRNA expression. The threshold standard deviation for intra- and inter-assay replicates was 0.3. PCR amplification efficiencies (E) were calculated for each miRNA and Taqman miRNA assay using the formula E  =  (10–1/slope− 1) × 100, using the slope of the semi-log regression plot of C_q_ versus log input of cDNA (10-fold dilution series of five points). A threshold of 10% above or below 100% was adopted. C_q_ values were scaled to highest expressing sample and normalized to previously validated miRNA controls (*miR-16 and miR-425)* [20]*.* MiRNA expression was calculated by the comparative cycle threshold (ΔC_q_) method, using qbasePLUS software (Biogazelle, NV, Belgium). Persistent C_q_ value > 35 considered outside viable detection thresholds.

### Statistical analysis

Statistical analysis was performed using SPSS (IBM SPSS Statistics for Macintosh, v23.0., IBM). The Kolmogorov-Smirnov test for normality was conducted. Data were log transformed (log_10_) for analysis when non-normal distribution was identified. Significance and associations of circulating miRNA levels were determined using the Mann-Whitney U test, t-test, ANOVA, Spearman’s Rho or Pearson correlation, as appropriate. Results with *p*-value < 0.05 were deemed to be significant. Binary logistic regression analysis was used and receiver operating characteristic (ROC) curves generated to evaluate the ability of chosen miRNAs to distinguish between metastatic and local breast cancer patients. This was performed both individually and for combinations of miRNAs.

### Ethics, consent and permissions

This study was conducted in accordance with the granted National University of Ireland Galway and University College Hospital Galway ethical approval. All patients clinic-pathological and demographic data were obtained from a prospectively maintained database.

## Results

### Circulating miRNA profiling of luminal a breast cancers and target selection

The discovery cohort identified 712 miRNAs (miR), of which 16 miRNAs were found to be significantly (*p* < 0.005) differentially expressed, between the metastatic and locally confined Luminal A breast cancer groups (Table 3). Three of these miRNAs were chosen for further validation (miR-181a, miR-329 and miR-331), selected based on evidence in the literature of their involvement in metastatic processes (Table 4). A fourth miRNA (miR-195) was chosen for investigation, based on a minimum 1.5 fold change in expression in the discovery cohort, and our previous work demonstrating miR-195 as a circulating biomarker in breast cancer [16].

**Table 3:** Top 10 differentially expressed miRNA, between local and metastatic breast cancers, in discovery cohort

Rank	miRNA	baseMean	log2FoldChange	lfcSE	stat	*P* value
1	hsa-miR-487a-5p	10.204322	−6.5949809	2.08622397	−3.1612046	0.00157118
2	hsa-miR-376c-3p	9.66930256	−6.2249402	2.16041009	−2.8813697	0.00395951
3	hsa-miR-181a-2-3p*	10.0812243	6.10481296	2.17498667	2.80682775	0.0050032
4	hsa-miR-6721-5p	11.0766671	−5.5985336	2.00252808	−2.7957329	0.00517822
5	hsa-miR-329-3p*	40.6587497	−4.2606132	1.5758723	−2.7036539	0.00685817
6	hsa-miR-665	4.33646293	−5.5104353	2.17214494	−2.5368635	0.01118505
7	hsa-miR-331-3p*	9.380562	−4.2942354	1.79092278	−2.3977781	0.01649485
8	hsa-miR-4433a-5p	15.8475709	3.90544954	1.65092455	2.36561359	0.01800022
9	hsa-miR-2277-3p	3.59270664	−4.7490645	2.08789239	−2.2745734	0.02293153
10	hsa-miR-6734-5p	25.3078788	3.92134038	1.73154349	2.26465024	0.02353415

*Selected miRNAs in **bold**

**Table 4:** Candidate miRNAs implicated in the metastatic cascade

miRNA	Metastatic process	Expression Pattern	Cancer
Mir-181a	EMT Migration and Invasion	**↑** **↑**	Breast [48] Colorectal [44] Breast [49]
Mir-329	Proliferation and migration Apoptosis	**↓** **↓** **↓**	Neuroblastoma [50] Gastric [51] Lung [52]
Mir-331	Proliferation and EMT	**↑**	Liver [37]

### Target miRNA as metastatic or local breast cancer biomarkers

The selected targets were validated on RNA extracted from the whole blood from 3 cohorts of patients, locally confined Luminal A breast cancer (*n* = 31), metastatic breast cancer (*n* = 22) and healthy controls (*n* = 21).

**Fig. 1:**
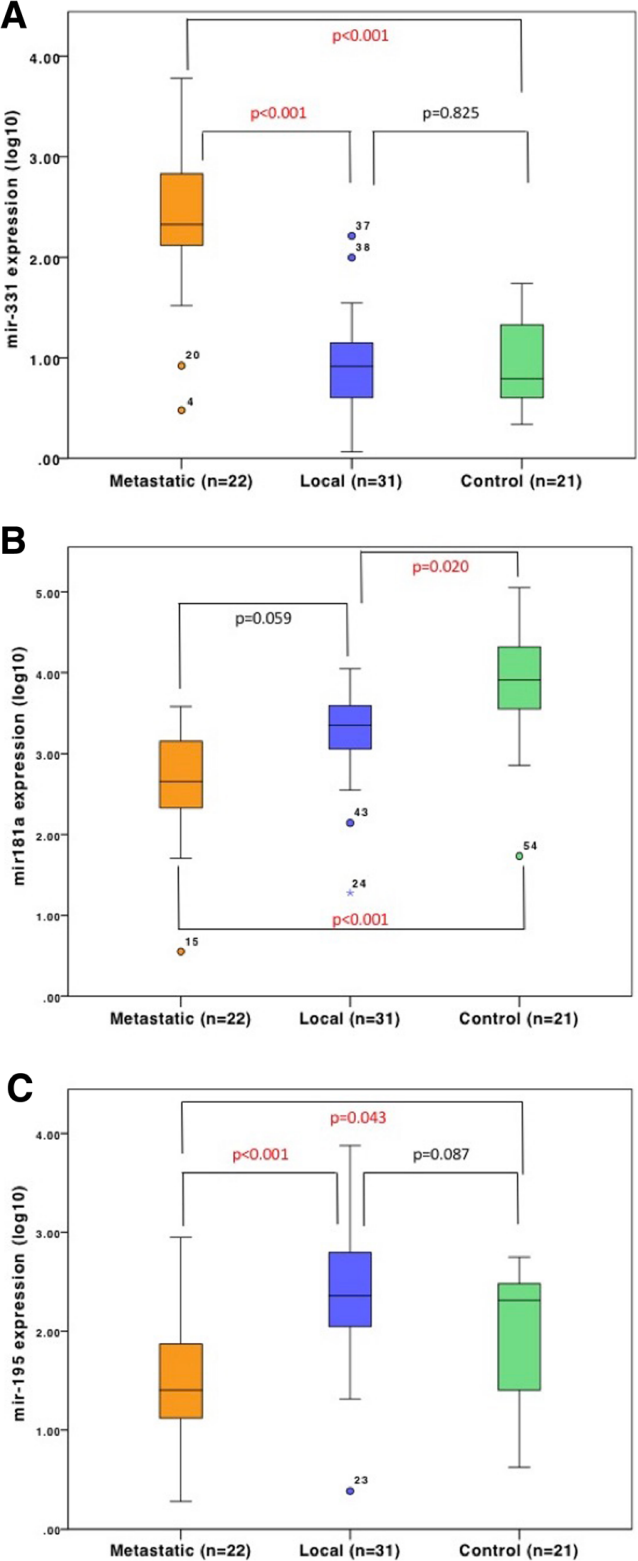
Target miRNA expression in Luminal A breast cancer groups and controls. RT-qPCR of target miRNA in indicated groups; locally confined Luminal A breast cancer (Local) (*n* = 31); metastatic breast cancer (Metastatic) (*n* = 22) and healthy controls (Control) (*n* = 21). **a** miR-331 expression. **b** miR-181a expression. **c** miR-195 expression. *p* < 0.05 considered significant. *p* values indicated on figure

Expression of miR-331 was significantly higher in the metastatic group compared to both the locally confined breast cancer group (*p* < 0.001) and the healthy control group (*p* < 0.001), corresponding to an average fold-change of 2.58 and 2.94 respectively. There was no significant difference in miR-331 expression between the locally confined breast cancer group and the control group (*p* = 0.825) (Fig. 1a).

Investigating miR-181a, its expression was significantly higher in the healthy control group in comparison to the metastatic group (*p* = 0.001), or the locally confined breast cancer group (*p* = 0.02), with an average fold-change of 1.4 and 1.19 respectively (Fig. 1b). Expression of miR-181a was lower in the metastatic group, compared to the local group (*p* = 0.059).

Examining expression of miR-195, it was significantly lower in the metastatic group compared to both locally confined breast cancer (*p* < 0.001) and healthy control groups (*p* = 0.043), with average fold-changes of 0.6 and 0.73 respectively (Fig. 1c). Interestingly, there was no significant difference in miR-195 expression between the locally confined breast cancer and the healthy control group (*p* = 0.087).

mir-329 was detectable in only 2 of the metastatic cohort (*n* = 13; with a mean C_q_ of 34.95) and detectable in 11 of the local cohort (*n* = 17; mean C_q_ 35.9) (data not shown). Due to the variability of this expression/detection further analysis of miR-329 was not performed.

### Target miRNA as breast cancer biomarkers

**Fig. 2:**
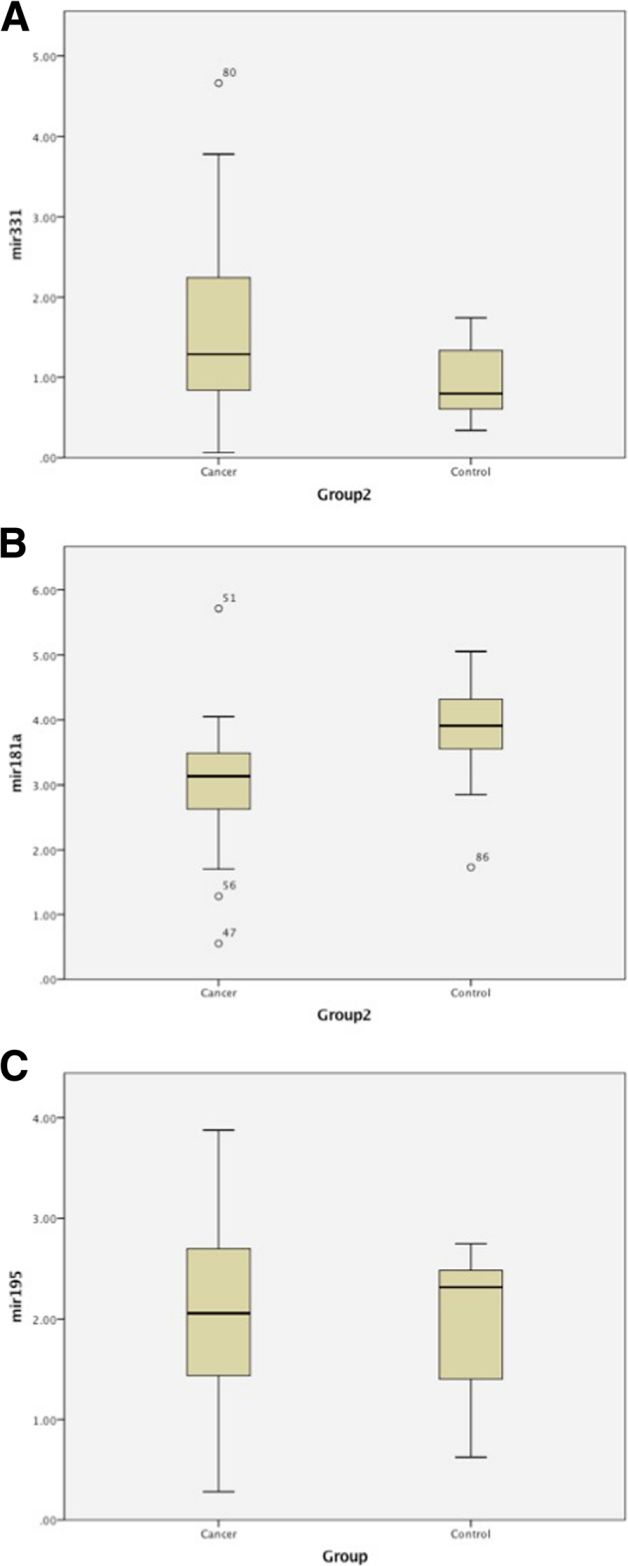
Target miRNA expression in breast cancer. RT-qPCR of indicated target miRNA in indicated groups. Breast Cancers (Cancer) (*n* = 53), Controls (n = 21). **a** miR-331. **b** miR-181a. **c** miR-195. *p* < 0.05 considered significant. p values indicated on figure

Pooling metastatic and local cancer groups together (*n* = 53) there was a significantly higher expression of miR-331 (*p* < 0.001) in cancer samples, compared to healthy controls (Fig. 2 a). The cancer group had a significantly lower expression of miR-181a (*p* < 0.001) (Fig. 2b). There was no significant difference for miR-195 (0 = 0.806) (Fig. 2c).

### Correlating target miRNA with clinicopathological features

**Fig. 3:**
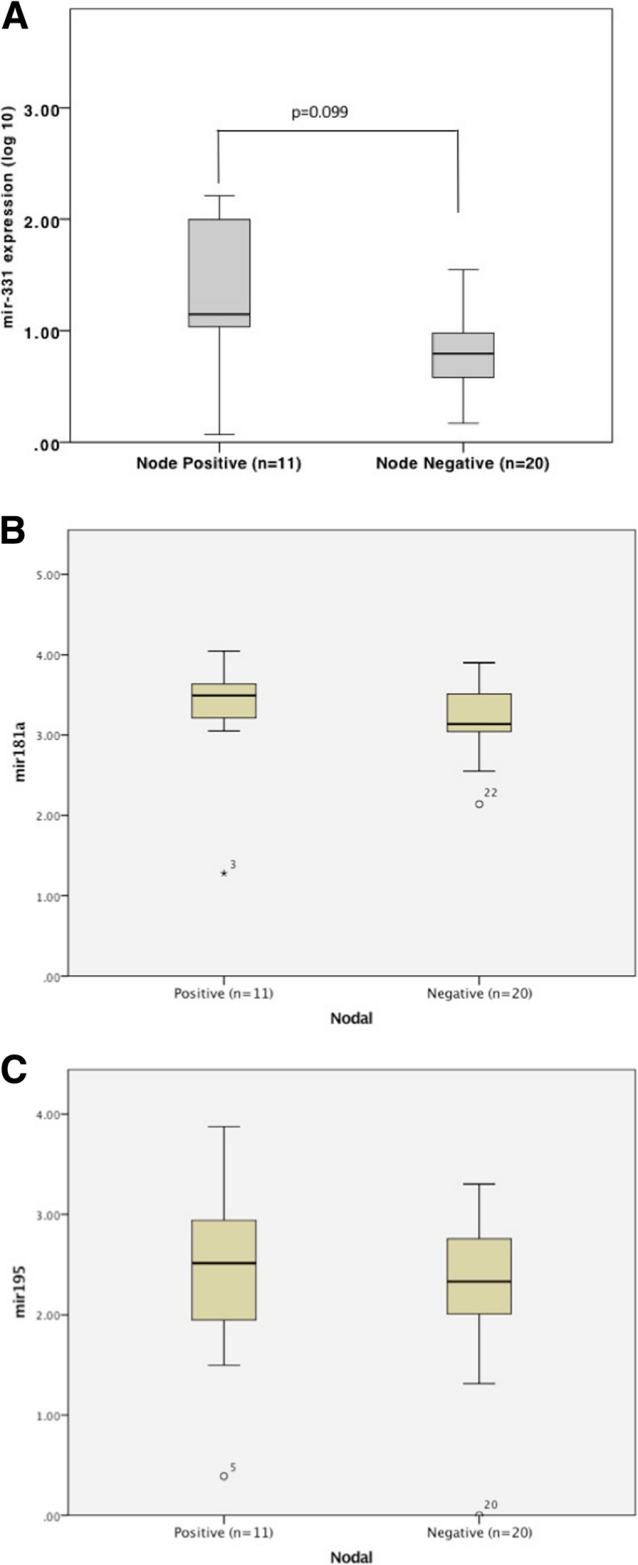
Target miRNA expression in breast cancers, by node status. RT-qPCR of indicated target miRNA in cancers, by nodal status (positive or negative). **a** miR-331. **b** miR-181a. **c** miR-195. *p* < 0.05 considered significant. *p* values indicated on figure

**Fig. 4:**
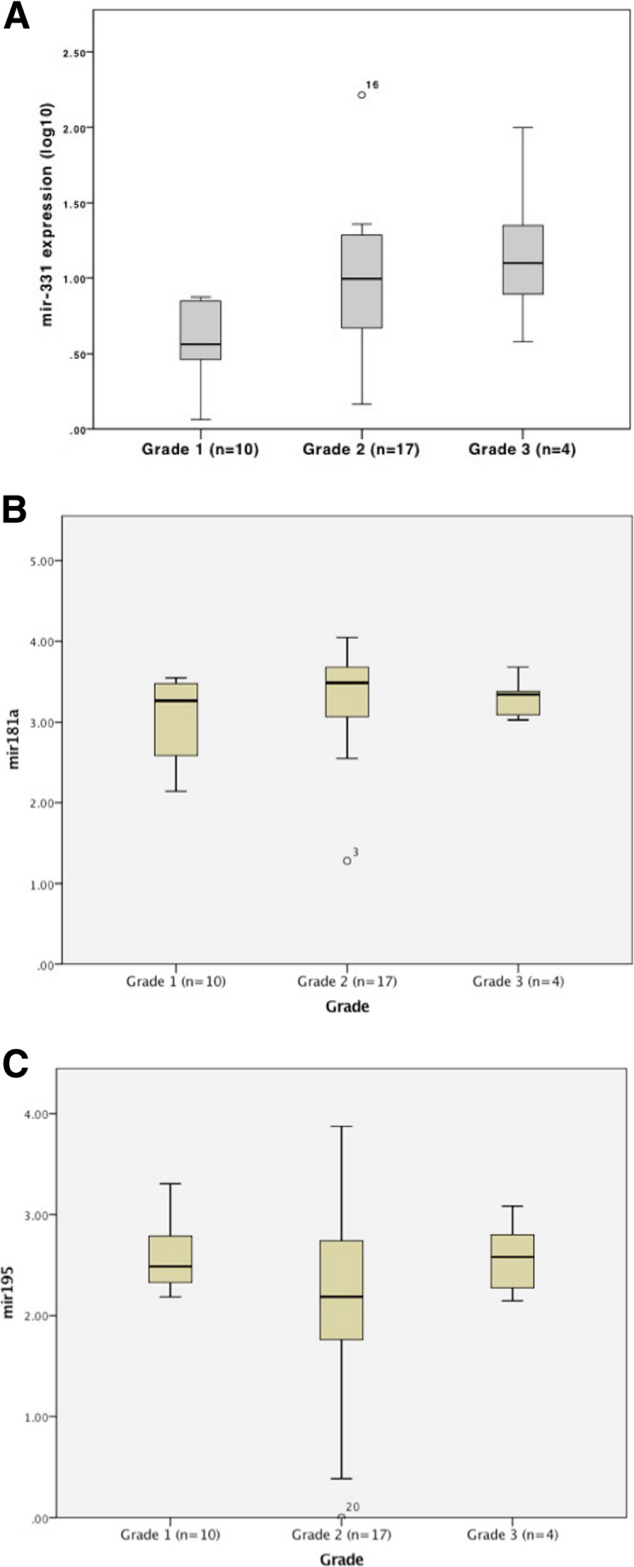
Target miRNA expression, by breast cancer grade. RT-qPCR of indicated target miRNA in cancers, by breast cancer grade: I (*n* = 10), II (*n* = 17), III (*n* = 4). **a** miR-331. **b** miR-181a. **c** miR-195. *p* < 0.05 considered significant. *p* values indicated on figure

The association of the target miRNA expression with key clinically relevant clinicopathological features was examined. Investigating expression in locally confined breast cancer group (*n* = 31) comparing lymph node positive disease (*n* = 11) to lymph node negative disease (*n* = 20) there was no significant difference for miR-331 (*p* = 0.099), miR-181a (*p* = 0.7) or miR-195 (*p* = 0.674) (Fig. 3a-c). There was no significant difference found between tumour grades (I-III) for miR-331 (*p* = 0.274), miR-181a (p = 0.6) or miR-195 (*p* = 0.37) (Fig. 4a-c). However, previous work demonstrated that changes in miR-195 expression correlated with disease stage (1–4) [16].

No significant difference between lymphovascular invasion (LV invasion) status (positive/negative) was observed for miR-331 (*p* = 0.31), miR-181a (p = 0.3) or miR-195 (*p* = 0.79) (Additional file 1: Figure S1A-C). Exploring any correlation between the target miRNA expression and tumour size, no significant correlation was found for miR-331 (*p* = 0.233), miR-181a (*p* = 0.942) or miR-195 (*p* = 0.175) (Additional file 1: Figure S2A-C).

### miRNA signature as a biomarker of metastatic luminal a breast cancers

**Fig. 5:**
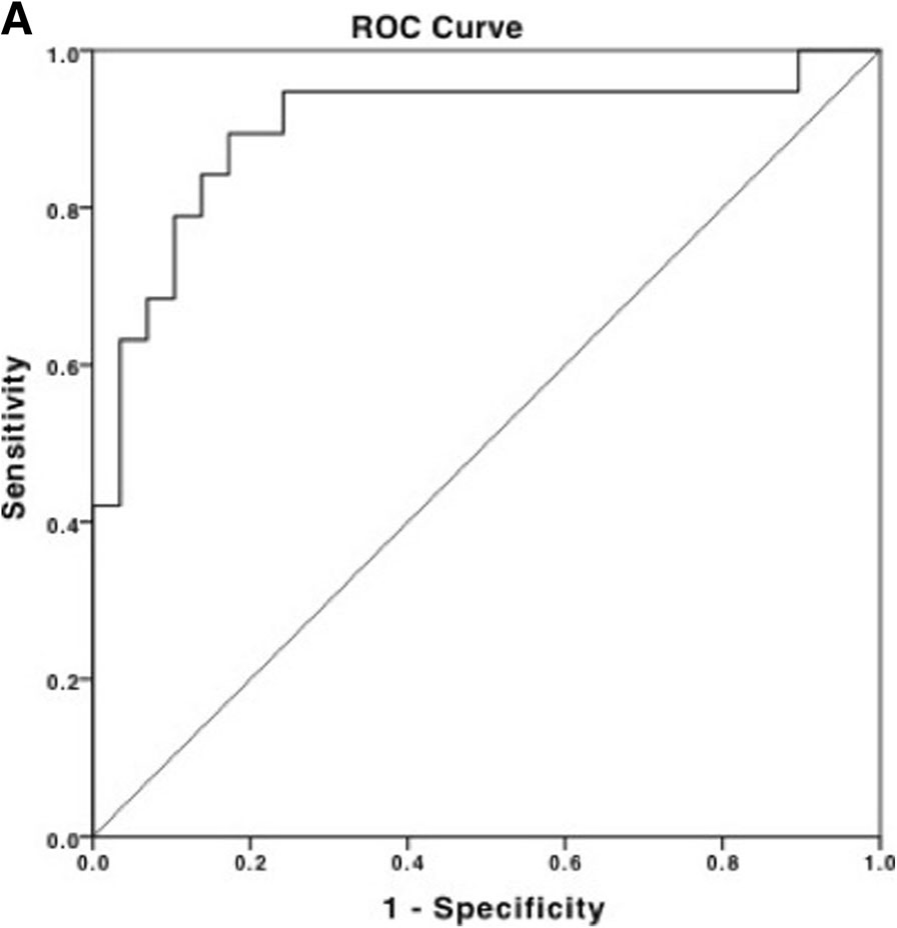
miRNA signature significantly distinguishes local from metastatic Luminal A breast cancer. **a** The signature of miR-331 and miR-195 distinguish metastatic from local breast cancer (AUC = 0.902)

A logistic regression was performed to ascertain the combined ability of the target circulating miRNAs miR-195, miR-181a and miR-331 to distinguishing metastatic from local disease. Analysing each individual miRNA, and the combination of miRNAs, the area under the curve (AUC) produced from receiver operator characteristic (ROC) curve generation using binary logistic regression was compared. The highest AUC of 0.902 was achieved combining miR-331 and miR-195, providing a sensitivity of 95% and a specificity of 76% (Fig. 5). The logistic regression model was significant [x^2^ (4)=28.98, *p* < 0.001]. Combining miR-181a with miR-195 [AUC of 0.86 (*p* = 0.35)] or miR-331 [AUC of 0.88 (*p* = 0.174)] did not contribute significantly to any biomarker signature (ROC Curves: Additional file 1: Figure S3A-B).

## Discussion

Despite considerable investment into the development of biomarkers and advances in our understanding of the underlying molecular landscape of breast cancer, only three established biomarkers (ER, PR, HER2) are mandatorily screened for in all newly diagnosed breast cancer patients. While these markers categorise breast cancer (into standardised, clinically relevant subtypes) and predict response to treatment, there remains a need for further biomarkers to better stratify high-risk patients and to monitor for the development of metastasis in real time.

A variety of clinical multigene/multiprotein tests capable of evaluating prognosis independent of traditional prognostic factors (such as grade and size) and are commercially available. Oncotype DX [21, 22], Mammaprint [23] and urokinase plasminogen activator (uPA)/PAI-1 [24, 25] have been evaluated in terms of their clinical utility in randomised prospective trials. These multi-analyte tests require invasive collection of tumour tissue, and their use is limited to informing treatment decisions for early stage breast cancers.

Non-invasive biomarker testing which can identify disease progression is of greater clinical value through easy, rapid access to samples, which can allow improved monitoring and the early identification of metastatic breast cancer. Traditional circulating markers include CA 15–3, CA 125 and CEA. While these have not been recommended for serial measurement by ASCO or ESMO [26, 27], increasing levels of these markers in breast cancer patients have been shown to precede the development of metastases, and in conjunction with prompt appropriate imaging can lead to improved therapeutic interventions, and patient outcomes [28, 29].

A number of new circulating biomarkers of metastatic disease have been investigated in recent years. Circulating tumour cells (CTCs) are quantifiable in blood and detection of > 5 (per 7.5 ml of whole blood) is associated with increased baseline levels of metastatic niches, and is an independent prognostic factor of relapse [30]. Cell free circulating tumour DNA (ctDNA) contains cancer specific somatic mutations and can be detected using qRT-PCR [31]. Detection of ctDNA in plasma following curative treatment can precede the clinical diagnosis of breast cancer metastasis with high accuracy [32, 33]. However, both of these require relatively large sample volumes to accurately detect tumour cells/DNA into the blood and they are not suitable for monitoring treatment response (compared to disease occurrence/presence). Additionally, CTC’s detection requires expression of known and sometimes variable markers (with high quality antibodies needed for the profiling). CTC or ctDNA genomic profiling needs known genomic changes and the support of high quality interpretation and analysis services to make sense of the data [34]. In contrast, miRNA can be detected in small volumes of blood, analysis is simple, and importantly they and can be used to directly monitor tumour responses to treatments.

As we enter the era of tailored breast cancer management clinicians require multiple tests, allowing disease detection/screening, aiding in treatment choice, and to monitor disease response, ultimately to achieve the optimal outcome for patients. Circulating miRNAs are an appealing adjunct to conventional diagnostic and prognostic modalities, as they are stable in circulation, easily quantifiable and can reveal further information of the underlying biology of the tumour. The potential of miRNAs to contribute to a “liquid biopsy” has been the focus of much research in recent years [35]. In this study 2 novel miRNA (mir-331 and miR-191a) and (expanding on our previous work [16]) mir-195 were validated as markers of distant metastatic Luminal A breast cancer, compared to patients with locally confined Luminal A breast cancer or age-matched healthy controls.

Investigating the molecular role of each of these validated miRNA, we find that confirmed targets of Mir-331 include HER2, HOTAIR, E2F1 and DOHH, with established links to metastatic processes such as cell proliferation, evasion of apoptosis, angiogenesis and EMT [36]. A recent study investigating mir-331 in hepatocellular carcinoma demonstrated that high expression of mir-331 associated with poor clinicopathological details and worse survival [37]. mir-331 was shown to target PHLPP (PH domain and leucine-rich repeat protein phosphatase) resulting in stimulation of protein kinase B (AKT) promoting EMT, proliferation and metastasis. Inhibition of mir-331 in vivo (in a xenograft mouse model using an anti-mir-331 vector) resulted in a marked inhibition of proliferation and metastasis, further supporting the designation of mir-331 as a tumour-promoting miRNA.

Mir-195 has previously been implicated as a diagnostic biomarker of breast cancer [16, 38], and has more recently been investigated as a tumour suppressor. Mir-195 has been shown to target Bcl-2, inducing apoptosis, and target FASN, HMGCR, ACACA and CYP27B1 in hormone-receptor positive breast cancer cell lines, suppressing tumour growth, EMT, invasion and metastasis [39, 40]. Other studies have shown that mir-195 regulates biological processes such as cell proliferation and cell cycle by targeting CDK4, CDK6, cyclin D1 and others [41–43].

miR-181a functions as a tumour suppressor, with re-expression of miR-181a inhibiting proliferation, migration, and invasion and promoting apoptosis (though regulation of Bcl-2) in cancer cells [44, 45]. Changes in miR-181a expression in AML tumours correlated with increased survival and improved treatments responses in a sub-cohort of patients [46]. Together these results point to a role for miR-181 in regulating tumour growth and metastasis in multiple cancers. While there are reports of divergent miRNA profiles between plasma and whole blood, our results support previous work confirming miR-195 expression in both [47]. It is likely that differences in miRNA expression seen are (at least partially) due to different collection methods and equipment, and the relative quantifiable expression levels retained after processing and RNA extraction.

Our results are encouraging and further advance the potential of circulating miRNAs as biomarkers that can contribute to breast cancer management. Further work is needed, using a larger, blinded multi-centre prospective study to fully validate our chosen miRNA as biomarkers of metastatic breast cancer. Our study was limited to ER positive, Luminal A breast cancer patients, and it is unclear if the dyseregulated miRNAs are subtype specific or if the same pattern of expression exists in all breast cancer subtypes. In the metastatic cohort our samples were taken at the time the patient had confirmed distant metastatic disease, and while our results suggest these miRNAs reflect the presence of metastasis, prospective collection of blood samples from patients with locally confined disease need to be conducted to confirm if the dysregulated miRNA expression preceded the development of metastatic disease. It is worth noting that the patient cohort investigated in this study were recruited almost entirely from the West of Ireland, potentially limiting the widespread applicability of these results. Additional studies are needed to investigate the potential clinical validity of these findings in other patient cohorts (multiple, independent and varied geographic and ethnic populations).

## Conclusion

Our study identified and validated two circulating miRNAs with differential expression able to separate metastatic from locally confined Luminal A breast cancer. Our results demonstrate that patients with metastatic disease have a higher expression of mir-331 and a lower expression of mir-195 in their circulation. In combination, these markers form a signature able to distinguish metastatic from local breast cancer with a high sensitivity and specificity. While miR-195 has previously been investigated as a suppressor of metastatic disease in breast cancer [40], to our knowledge this is the first study to identify mir-331 as a potential promoter of breast cancer metastasis. Further research is required to elucidate the molecular mechanisms/activity of miR-331 and to establish if miR-331 dysregulation contributes to the development of metastatic disease, of if it is a consequence of the metastatic changes. Our work contributes to the evolving understating of the molecular dysregulation in breast cancer and provides biomarkers that may contribute to the optimization of breast cancer management.

## Additional file

Additional file 1: **Figure S1.** Target miRNA expression in breast cancer, by lymphovascular invasion (LV invasion) status. **A.** miR-181 expression. **B.** miR-331 expression. **C.** miR-195 expression**.** Note: expression of miR-329 was below/outside detection threshold, with persistent Cq value > 35 in over 50% of samples. **Figure S2**. miRNA expression in breast cancer does not correlate with tumour size. **A.** miR-181 expression. **B.** miR-331 expression. **C.** miR-195 expression. **Figure S3.** miRNA signature combinations that did not significantly distinguish local from metastatic Luminal A breast cancer. ROC curves: **A.** miR-181 and miR-195. **B.** miR-331 and miR-181. (PDF 611 kb)

## Abbreviations

ER: estrogen receptor
FISH: fluorescence in situ hybridization
HER2: Her2 over-expressing breast cancer
Her2: human epidermal growth factor receptor 2
HR: hormone receptor
HR: hormone receptor
IHC: immunhistochemical
OS: overall survival
PR: progesterone receptor
PRS: post-recurrence survival
RT-qPCR: real-time quantitative reverse transcriptase PCR
TN: Triple Negative
