# Supplementary figures and images for: Circulating microRNAs miR-331 and miR-195 differentiate local luminal a from metastatic breast cancer
Source: BMC Cancer. 2019 May 10;19:436. doi: 10.1186/s12885-019-5636-y (PMC6511137; doi:10.1186/s12885-019-5636-y)

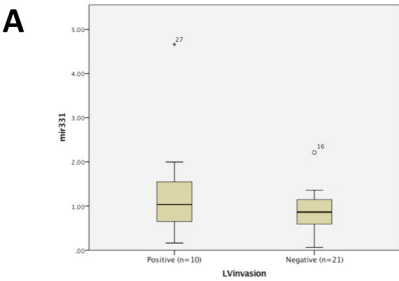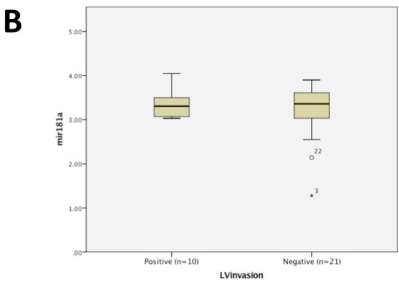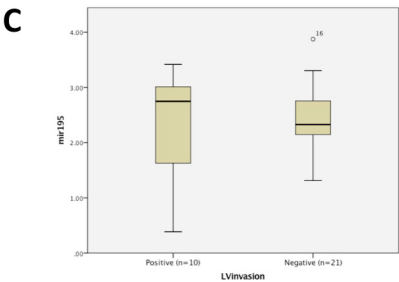

**Supplemental Figure 1.**

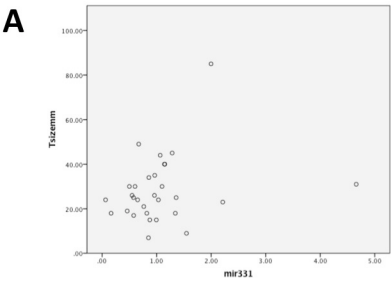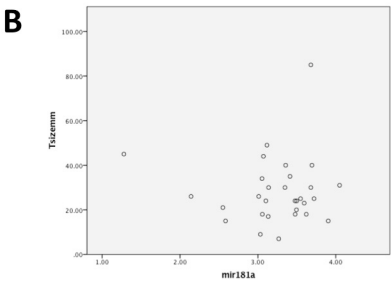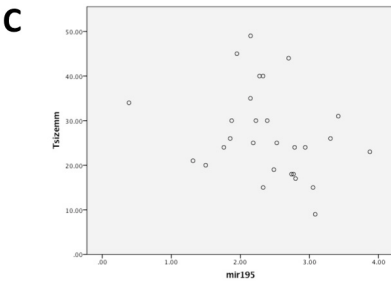

**Supplemental Figure 2.**

A

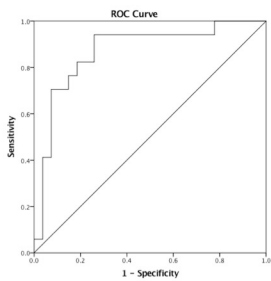

miR-195 and miR-181a

B

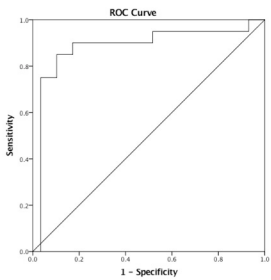

miR-331 and miR-181a

Supplement: Supplementary file 1 — Figure S1. Target miRNA expression in breast cancer, by lymphovascular invasion (LV invasion) status. A. miR-181 expression. B. miR-331 expression. C. miR-195 expression. Note: expression of miR-329 was below/outside detection threshold, with persistent Cq value > 35 in over 50% of samples. Figure S2. miRNA expression in breast cancer does not correlate with tumour size. A. miR-181 expression. B. miR-331 expression. C. miR-195 expression. Figure S3. miRNA signature combinations that did not significantly distinguish local from metastatic Luminal A breast cancer. ROC curves: A. miR-181 and miR-195. B. miR-331 and miR-181. (PDF 611 kb) [file 12885_2019_5636_MOESM1_ESM.pdf]
